# 中国髓外浆细胞瘤诊断与治疗专家共识（2024年版）

**DOI:** 10.3760/cma.j.cn121090-20231107-00253

**Published:** 2024-01

**Authors:** 中华医学会血液学分会浆细胞疾病学组 中国医师协会多发性骨髓瘤专业委员会

**Keywords:** 浆细胞瘤, 诊断，鉴别, 临床方案, Plasmacytoma, Diagnosis, differential, Clinical protocols

## Abstract

髓外浆细胞瘤（extramedullary plasmacytoma, EMP）是恶性浆细胞病的一种特殊类型，表现复杂、异质性强。大部分EMP预后差，缺乏循证医学证据支持下的预后分层系统和理想的治疗策略，无法满足临床需求。为提高对这类疾病的认识，中华医学会血液学分会浆细胞疾病学组和中国医师协会多发性骨髓瘤专业委员会编写《中国髓外浆细胞瘤诊断与治疗专家共识》，旨在规范EMP的临床诊治，最终改善浆细胞瘤患者的总体生存。

## Abstract (en)

Extramedullary plasmacytoma (EMP) is a special type of malignant plasmacytosis, which is complex and heterogeneous. Most EMP patients have poor prognosis and lack a stratified prognostic system or ideal treatment strategy supported by evidence-based medical evidence, which cannot meet clinical needs. In order to improve the understanding of this disease entity, Plasma Cell Disease Group, Chinese Society of Hematology, Chinese Medical Association and Chinese Myeloma Committee-Chinese Hematology Association developed the “Chinese Expert Consensus on the diagnosis and treatment of extramedullary plasmacytoma”, which aims to standardize the clinical diagnosis and treatment of EMP and ultimately improve the overall survival of patients with plasmacytoma.

一、概述

髓外浆细胞瘤（extramedullary plasmacytoma，EMP）是恶性浆细胞病的一种特殊类型，指发生于骨髓外的浆细胞恶性肿瘤，这类疾病最早在20世纪40年代报道。EMP表现复杂、异质性强。目前对于EMP的定义和名称尚无统一标准，大部分EMP预后差，也缺乏循证医学证据支持下的预后分层系统和理想的治疗策略，无法满足临床需求。迄今为止国内外尚无针对EMP的专家共识，为了提高对这类疾病的认识，中华医学会血液学分会浆细胞疾病学组和中国医师协会多发性骨髓瘤专业委员会汇集各专家智慧，基于有限的证据，编写此共识，旨在规范EMP的临床诊治，最终改善浆细胞瘤患者的总体生存。

二、EMP的分类、流行病学、临床表现和预后

EMP可根据是否达到多发性骨髓瘤（MM）的诊断标准，分为孤立性浆细胞瘤（solitary plasmacytoma，SP）和多发性骨髓瘤髓外浸润（extramedullary multiple myeloma，EMM）。前者可根据受累部位、是否伴随骨髓侵犯进一步分类；后者可根据受累部位、髓外病灶出现的时机等进行分类。具体分类见图1。

**图1:**
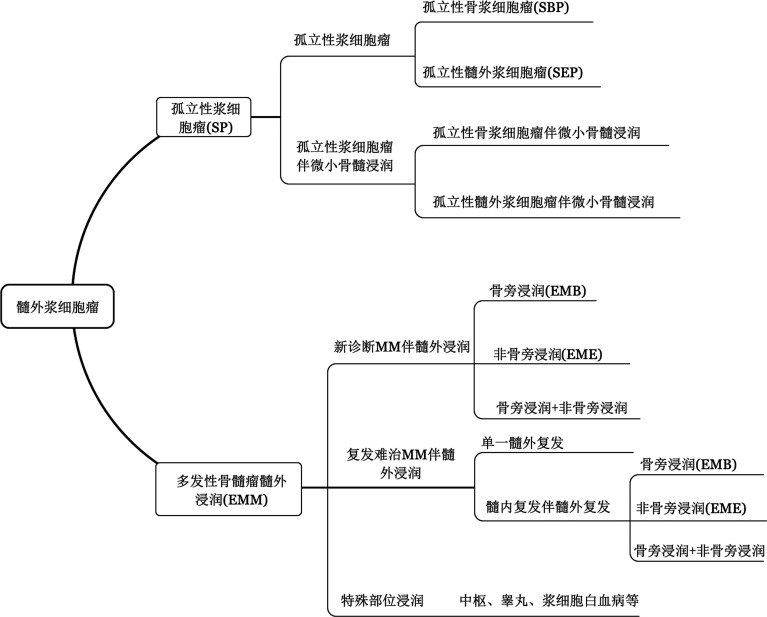
髓外浆细胞瘤的分类

（一）孤立性浆细胞瘤

孤立性浆细胞瘤指单个病灶的浆细胞瘤[1]。单个骨骼的浆细胞瘤称为孤立性骨浆细胞瘤（solitary bone plasmacytoma，SBP），部分患者可能伴有孤立骨病灶周围软组织的延伸受累，这种情况也属于SBP范畴。也有人将之命名为solitary plasmacytoma of bone（SPB）。为统一命名，本共识将孤立性骨浆细胞瘤伴或不伴周围软组织延伸统一简称为SBP。单个软组织或器官的浆细胞瘤，称为孤立性髓外浆细胞瘤（solitary extramedullary plasmacytoma，SEP），亦有人命名为骨外浆细胞瘤（extraosseous plasmacytoma），为统一命名，本共识将孤立性髓外浆细胞瘤统一简称为SEP。根据骨髓中是否存在克隆性浆细胞增殖，可进一步分为孤立性浆细胞瘤或孤立性浆细胞瘤伴微小骨髓浸润（solitary plasmacytoma with minimal marrow involvement），后者指骨髓中可以检测到的极少克隆性浆细胞，但比例低于10％，未达到MM诊断标准。

1. 流行病学：孤立性浆细胞瘤属于罕见病，占所有浆细胞疾病约 5％，以 SBP 多见。SBP占所有 SP 病例的 70％，男女比例约为 2∶1，欧美国家发病率为 0.09～0.19/10 万人年，中位诊断年龄为 55～65岁[2]。SEP 占所有孤立性浆细胞瘤病例的 30％，男性较常见，中位诊断年龄为 55～60岁，发病率为 0.063～0.10/10 万人年[2]。

2. 临床表现：

（1）SBP 可累及任何骨骼，但最常见中轴骨（占83.1％～84.3％），尤其是脊椎；四肢骨发生率为15.7％～16.9％[2]–[3]。主要表现是受累部位的骨痛、肿胀、病理性骨折等，部分患者早期无症状，仅通过影像学检查发现。

（2）SEP最常位于头颈区域（73％～76％），特别是上呼吸道，包括鼻腔、口咽、唾液腺及喉部；也可见于胃肠道（3.6％～7.4％）、下呼吸道（7％）、淋巴结和脾脏（4.6％～5.3％）、皮肤和皮下区域（5.5％）、内分泌腺（3％）以及其他罕见部位，包括睾丸、膀胱、尿道、卵巢等[4]–[5]。大多数患者的症状与肿块位置相关，部分患者早期无症状。

（3）SBP和SEP均无浆细胞疾病引起的贫血、高钙血症、肾功能不全或溶骨性改变（SBP 本身受累部位除外）。60％～70％的SBP患者和<25％的SEP患者在血清和（或）尿液中可检测到少量单克隆免疫球蛋白，部分患者在骨髓中可检测到<10％的克隆性浆细胞。

3. 预后：

（1）SBP 10年进展为MM的比例为43％～100％，10年总生存（OS）率为33％～84％，在诊断时有以下指标往往提示预后不良[6]：①骨髓有克隆性浆细胞浸润的证据；②单克隆免疫球蛋白水平>5 g/L；③血清游离轻链比例超出正常范围（<0.26 或>1.65）；④治疗后血清单克隆免疫球蛋白水平持续≥5 g/L。

（2）SEP 预后优于 SBP，进展为MM 的比例明显降低，10年进展为MM的比例为5％～36％，10年 OS率为56％～94％。头颈部受累者预后较好[7]。

（二）多发性骨髓瘤髓外浸润

在大多数MM患者中，浆细胞增殖仅局限于骨髓腔内，但部分患者可生长在骨髓外，称为多发性骨髓瘤髓外浸润。主要见于两种方式[8]：①肿瘤破坏骨皮质后直接向骨髓外生长，称为骨旁浸润（extramedullary bone related，EMB），亦有称为 paraskeletal with tumor masses arising from skeletal lesions（PS）或 paraosseous plasmocytoma（PO）。为统一命名，本共识将骨旁浸润的EMM统一简称为EMB。②肿瘤脱离骨髓微环境，经过血行播散后在软组织或器官中生长，不与骨骼结构接触，称为非骨旁浸润（extramedullary extraosseous，EME），亦有称为extramedullary disease（EMD）或extramedullary soft tissue（EMS）。为统一命名，本共识将非骨旁浸润的EMM统一简称为EME。部分MM患者可以同时出现EMB和EME，这种情况，由于EME预后更差，诊断和治疗上按EME处理。EMM还可以根据发生的时机分为新诊断多发性骨髓瘤（newly diagnosed multiple myeloma，NDMM）伴髓外浸润（或称原发髓外浸润）和复发难治多发性骨髓瘤（relapsed or refractory multiple myeloma，R/R MM）伴髓外浸润（或称继发髓外浸润）。

EMM的发生机制目前未明确，目前已知的有以下几种可能机制。①各种细胞因子和趋化因子的改变，促使肿瘤细胞生长不再需要依赖于骨髓微环境，从而向骨髓外扩散，如趋化因子受体和黏附分子表达量降低、CD81/CD82低表达、肝素酶高表达、生长因子和缺氧导致CXCR4上调等，这些改变使骨髓瘤细胞获得了EMM表型，容易发生髓外浸润[9]。②基因组结构变异：有报道EMM患者中有80％发生MAPK通路突变；20％存在高频突变，其中40％存在TP53缺失；40％获得继发性MYC/FGFR3/CCND2易位。此外，EMM患者还容易发生3q扩增、1q扩增、1p缺失、13q缺失等，这些分子改变促使肿瘤细胞容易发生髓外浸润[10]。③肿瘤细胞增殖、糖酵解和氧化磷酸化增加，提高肿瘤细胞的自主增殖能力[11]。④细胞毒性T细胞和NK细胞抑制分子上调，促使肿瘤细胞发生免疫逃逸并向骨髓外迁移[12]。⑤骨髓微环境中的基质细胞与骨髓瘤细胞相互作用，增加细胞迁移能力和细胞因子信号传导，从而促进骨髓瘤细胞髓外浸润[13]。

髓内生长和髓外生长的浆细胞存在较大的差异，在不同部位的髓外病灶也有空间异质性。EMM髓外病灶的增殖指数Ki-67抗原表达显著高于髓内浆细胞，且髓外浆细胞CD56和CD38表达水平降低。髓外浆细胞17p−、t（4;14）和1q21扩增发生频率也更高。因此，骨髓内的肿瘤细胞遗传学和分子生物学改变不能反映EMM的真实情况，建议行病灶及相关组织物活检。

随着MM患者生存期的延长、对EMM认识的提高、以及正电子发射断层显像/X 线计算机体层成像（PET/CT）各种更精准的检测技术等的广泛应用，EMM的检出率在逐渐增加[14]。20世纪报道EMM在MM中报道发生率仅为4％～6％，而本世纪以来，EMM的发生率则逐渐升高。其中，新诊断EMB在MM中报道检出率通常超过 10％，复发难治EMB的检出率更是可以高达30％以上。新诊断EME在MM中检出率为1.75％～4.80％，复发难治EME的检出率为3.4％～13.1％[15]。检出率的不同受到多种因素的影响，包括定义、检测手段以及不同的人群等。

1. 临床表现：EMM的临床表现主要包括MM的临床表现和髓外病灶的表现。前者可参照MM相关指南[16]，但其病情趋向于快速，有些与受累部位相关。其中EMB常见累及部位为脊椎（57％～78％）和肋骨（46.1％～48.0％），其他的部位还包括胸骨、颅骨、骨盆等。EME常见累及部位为皮肤或软组织（23％～40％）、淋巴结（10.2％～17.3％）、腹膜后脏器（4.0％～27.3％）、肺部及胸膜（3.6％～15.0％）、肝脏（5.8％～9.3％）、中枢神经系统（3.6％～10.1％）和生殖系统（1.8％～5.0％）。大多数患者（65％）仅有 1 个病灶出现，35％ 的患者存在多个病灶。

2. 预后：EMM患者若只行骨髓内肿瘤细胞的细胞遗传学检查，往往会因肿瘤细胞少而趋于阴性；且骨髓内外肿瘤细胞差异性较大，导致髓内细胞遗传学改变不能真实反映EMM预后；又由于早期EMM的β_2_-微球蛋白不高，白蛋白不低，ISS/R-ISS 分期不适用于EMM患者。

EMM是MM的独立不良预后因素。EMM的预后与类型、肿物大小、数量、细胞遗传学特征等相关。总体而言，EME的预后比EMB更差，复发难治EMM特别是EME是目前MM治疗领域的难题。初治EMB的OS期为28.8～63.5个月，初治EME的OS期为19.2～30个月，复发难治EMB的OS期为16～28.8个月，复发难治EME的OS期为7～19.2个月[15]。

髓外病灶单个直径超过5 cm、个数大于2个均是EMM的不良预后因素。一些特殊部位的EMM预后极差，如中枢神经系统、胸膜、睾丸等。此外，髓外病灶存在高危细胞遗传学特征及 Ki-67指数高的患者预后不良。

（三）多发性骨髓瘤中枢神经系统浸润

多发性骨髓瘤中枢神经系统浸润是一种罕见的髓外浸润，指通过常规细胞学和流式细胞术或直接组织取样和（或）影像学（MRI、CT或PET-CT）证实的软脑膜、硬脑膜或脑实质受累，可以伴或不伴神经系统相关异常症状[17]。中枢神经系统浸润占所有MM的1％～6％，男女发病率类似，中位发病年龄53岁，预后极差，未经治疗的患者OS期为2个月，治疗的患者OS期为8个月[18]。

浆细胞白血病（plasma cell leukemia，PCL）是一种特殊类型的髓外浸润，侵袭性强，预后较差。我们将另文介绍。

三、EMP的诊断

（一）检查项目

1. 血液检查：血常规、肝肾功能（包括白蛋白、球蛋白、乳酸脱氢酶、血清肌酐）、电解质（包括钙离子）、凝血功能、β_2_-微球蛋白、血清免疫球蛋白定量、血清游离轻链、血清蛋白电泳（包括M蛋白含量）、血清免疫固定电泳。

2. 尿液检查：尿常规、24 h尿蛋白定量、尿 M 蛋白定量或尿轻链定量、尿免疫固定电泳。

3. 骨髓检查：骨髓细胞学涂片、流式细胞术［抗体参照《中国多发性骨髓瘤诊治指南（2022年修订）》[16]］、骨髓活检＋免疫组化（骨髓免疫组化建议应包括针对如下分子的抗体：CD19、CD20、CD38、CD56、CD138、κ轻链、λ轻链）、骨髓浆细胞FISH检测，参照《中国多发性骨髓瘤诊治指南（2022年修订）》。

4. 影像学检查：推荐全身低剂量 CT、全身 MRI 成像或全身PET-CT。其中，诊断EMM时 PET-CT 敏感度最佳。在评估中枢神经系统MM方面，MRI 比头颅CT具有更高的灵敏度。

5. 髓外病变部位的病理活检：推荐在新诊断或复发难治时对怀疑髓外病变的部位进行穿刺或手术活检，并完善免疫组化（检测分子项目推荐同骨髓活检，包括代表组织增殖指数的Ki-67）及细胞遗传学等检查。

（二）诊断标准

1. 孤立性浆细胞瘤：

（1）孤立性浆细胞瘤的诊断必须满足以下 4 条[19]：①活检证实的骨或软组织浆细胞瘤；②骨髓中没有克隆性浆细胞增殖的证据；③全身骨骼检查（推荐全身 PET-CT或全身MRI检查）未发现除原发孤立病灶外的其他病变；④无浆细胞疾病引起的终末器官损伤，包括SLiM CRAB［SBP本身受累骨质破坏部位除外详见《中国多发性骨髓瘤诊治指南（2022年修订）》］。

（2）孤立性浆细胞瘤伴微小骨髓病变的诊断必须满足以下4条[19]：①活检证实的骨（SBP伴微小骨髓浸润）或软组织/器官孤立性病变（SEP伴微小骨髓浸润）；②骨髓中有克隆性浆细胞增殖的证据，但浆细胞比例小于10％；③脊柱和骨盆的骨骼影像学检查（推荐全身PET-CT）未发现除原发孤立病灶外的其他病变；④无浆细胞疾病引起的终末器官损伤包括 SLiM CRAB（SBP本身受累骨质破坏部位除外）。

2. 多发性骨髓瘤髓外浸润：

多发性骨髓瘤髓外浸润诊断必须满足以下3条[20]–[21]：①必须满足 2014国际骨髓瘤工作组（IMWG）MM的诊断标准；② MRI 或 PET-CT 等影像学检查发现存在骨旁或其他软组织/器官的髓外病变；③病变组织部位活检存在浆细胞瘤。

四、EMP治疗建议

（一）孤立性浆细胞瘤（包括SBP和SEP）

1. 放疗：放疗是治疗孤立性浆细胞瘤的首选方案。推荐放疗剂量为35～50 Gy，周期为4～5周。对于<5 cm的肿瘤，可给予较低剂量（如 35～40 Gy）放疗；但对于>5 cm肿瘤，倾向给予较高剂量（如 40～50 Gy）放疗。放疗的局部缓解率超过 80％～90％，对直径<5 cm的肿瘤放疗效果更佳[22]。

2. 手术：SP通常不需要手术切除，在大多数情况下，手术切除主要是在紧急减压或病灶活检时进行。即使因诊断已将浆细胞瘤部分或完全切除，也应对肿瘤部位实施局部放疗以减少复发机会[3]。

3. 化疗：对于肿瘤直径>5 cm或对放疗反应差的患者，以及骨髓中出现克隆浆细胞等具有高风险特征的SP患者，也可考虑联合全身化疗和（或）auto-HSCT[23]。

（二）多发性骨髓瘤髓外浸润

1. 治疗目标：争取最大程度血液学反应和EMP缓解，改善器官的终末并发症，防止早期复发或早期死亡，最终延长生存。

2. 治疗方法或药物选择：当前可用于EMM的治疗药物包括：蛋白酶体抑制剂（protease inhibitors，PI）、免疫调节剂（immunomodulatory drugs，IMiD）、细胞毒药物、免疫治疗［如单克隆抗体、嵌合抗原受体T（chimeric antigen receptor T，CAR-T）细胞等］以及其他小分子靶向药物等，或鼓励患者参加新药临床试验。

（1）PI：①硼替佐米：硼替佐米是NDMM患者的一线用药，且具有广泛的组织渗透性。一个纳入8项硼替佐米或IMiD治疗NDMM患者（2 332例）临床研究的荟萃分析显示，EMM（267例，其中EMB患者243例）患者和无 EMM患者的中位无进展生存（PFS）期相似（25.3个月对25.2个月）[24]，该结论表明以硼替佐米为基础的治疗方案对NDMM伴EMM的患者有益，尤其是对EMB患者有益。认为含硼替佐米的诱导方案-auto-HSCT-维持联合局部放疗的整体方案已使EMB患者的生存与无EMM患者的生存相似。Mayo在 2017 年指南中也推荐含硼替佐米的方案治疗 EMM[25]。②卡非佐米：一项使用卡非佐米治疗45例患有EMM的R/R MM患者的回顾性研究中，髓外总体有效率（overall response rate，ORR）为 27％[26]，初步显示其在EMM中的作用。

（2）IMiD：①来那度胺：相关文献报道极少。可穿透血脑屏障，个案显示对于中枢神经系统受累的髓外浆细胞瘤有效。②泊马度胺：Short等[27]报道了13例EMM患者，经过硼替佐米、沙利度胺、来那度胺治疗后复发难治的EMM患者应用泊马度胺联合低剂量地塞米松治疗，2例患者达到完全缓解（CR），2例患者达到部分缓解（PR）。因此，含泊马度胺的方案可能在EMM中有一定作用。

（3）细胞毒药物：①传统化疗方案：Huynh等[28]回顾性分析了2006年至2018年采用PACE方案治疗 43 例R/R MM伴EMM的患者，ORR为 58％，中位 PFS 期为 5 个月，中位 OS 期为 9 个月。另有研究表明使用 VDT-PACE 方案及其类似方案治疗的 141 例R/R MM患者[29]，36％ 合并EMM，EMM与非EMM患者的ORR分别为57.1％和 52.9％，提示VDT-PACE方案可能改善髓外浸润患者不良预后的影响。Mayo发表的 R/R MM 治疗指南中推荐，对于伴继发性PCL或EMM的患者，若体能状况较好，可使用2个周期的VDT-PACE方案治疗以控制疾病，并尽可能通过自体或异基因造血干细胞移植巩固疗效[25]。②苯达莫司汀：2022年欧洲血液学年会曾报道一项苯达莫司汀、泊马度胺联合地塞米松（BPD）治疗 21 例R/R MM合并EMM患者的临床研究[30]，结果显示，经治疗后所有首次复发患者在治疗后ORR为 100％，3 例患者达到CR，7 例患者达到非常好的部分缓解（very good partial remission，VGPR）。另一个回顾性研究显示BPD治疗11例RRMM合并EMM，ORR为54％，2年PFS率为71.3％，2 年OS率为81.8％[31]。以上临床数据显示其对EMM有一定疗效。但需注意的是苯达莫司汀对干细胞有一定抑制作用，需序贯auto-HSCT的患者慎用。

（4）免疫治疗：①达雷妥尤单抗（Daratumumab，Dara）：Meral[32]报道了Dara-VCd 治疗新诊断以及首次复发的MM伴髓外病变患者的疗效和安全性。共纳入40例患者（72.5％为新诊断，27.5％为首次复发），65％为EME，45％为EMB（4 例患者既有EME也有EMB）。中位随访12个月，初步结果显示，CR率≥40％，中位PFS期为15.3个月。另一项前瞻性的Ⅱ期临床试验，纳入32例R/R MM伴EMM患者接受 Dara 联合DECP方案化疗[33]，结果显示，既往接受过中位 3 线治疗、100％硼替佐米暴露患者的ORR为67.7％，其中CR率为35.5％，且19.4％患者持续缓解。中位随访11个月，中位 PFS期和中位OS期分别为5个月和10个月。②CAR-T：CAR-T细胞疗法在既往接受多线治疗的R/R MM治疗上显示出非常好的疗效。近年来陆续有研究报道 CAR-T 细胞疗法对EMM的治疗效果显著。靶向B细胞成熟抗原（BCMA）的CAR-T制剂 bb2121（也称为 ide-cel）在伴髓外浸润的R/R MM中显示出良好的抗肿瘤活性[34]，EMM（50例）和非 EMM（78例）组的ORR分别为70％和76％，CR率分别为24％和39％，中位 PFS期分别为7.9个月和10.4个月。但一项荟萃分析的结果表明，BCMA CAR-T细胞疗法对伴有EMM的患者ORR高达78％，但在 360 d内，获得应答的EMM患者中有一半复发[35]。另有一项荟萃分析的结果也显示，虽然早期EMM组与非EMM组的ORR差异无统计学意义（*P*＝0.940），但EMM组的PFS期并未延长[36]。以上结果表明，CAR-T细胞疗法可以为EMM患者提供短期缓解，但长期疗效可能并不理想。但近期有个案报道使用LCAR-B38M CAR-T 治疗多线复发EMM 患者获得5年无病生存，因此CAR-T细胞疗法的长期疗效还有待观察[37]。

（5）其他小分子靶向药物：塞利尼索（Selinexor）：STORM试验中[38]，27 例基线伴髓外浆细胞瘤患者，其中 5 例患者髓外浆细胞瘤消失，2 例患者肿瘤缩小，2 例患者肿瘤代谢降低。该结果支持Sel-dex在EMM患者中具有活性。

（6）放疗：放疗是SP患者的标准治疗。但在MM患者中仅用于全身化疗后或化疗后残余单个病灶的补充治疗。目前较少有研究报道放疗对EMM的治疗效果。

（7）移植：目前关于auto-HSCT能否克服EMM的不良预后尚存在争议。对骨旁浸润的EMM患者，含硼替佐米的诱导方案-auto-HSCT-维持治疗并在移植后加局部放疗已使这些患者PFS和OS与无EMM患者无差别[39]。2005年至2014年间在欧洲血液和骨髓移植学会登记了3 744例接受单次或双次auto-HSCT的患者结局[40]，结果显示，接受auto-HSCT 治疗后，无EMM组和EMB组的3年PFS率分别为47.9％和50.0％，3年OS率分别为80.1％和77.7％，而EME患者的3年PFS率为39.9％，OS率为58.0％。上述结果表明，新药年代，auto-HSCT可克服EMB不良预后，尤其是髓外病变仅累及一个部位的患者，可通过auto-HSCT及移植后放疗获益，但EME患者预后仍较差。一项多中心回顾性研究 226例EMM（2010年至2017年），新诊断130例，复发 96例，结果显示EME也能从移植中获益，在接受auto-HSCT的患者中，PFS期为49个月（EMB 51.7个月，EME 46.5个月，差异无统计学意义）。对于没有接受移植的患者，中位PFS期为28.1个月（*P*<0.001），结果提示虽然EME患者接受auto-HSCT的预后仍较差，但仍然能从auto-HSCT中获益[41]。

对于双次 auto-HSCT是否能获益目前仍有争议。有研究显示与单次auto-HSCT相比，双次auto-HSCT并不能改善EMM患者的总体预后；allo-HSCT对EMM的作用目前仍缺乏证据，但allo-HSCT仍可能是EMM的一种潜在治疗选择[42]。

3. 治疗推荐：EMM患者的治疗方案需根据患者的年龄、体能状况和髓外病变情况制定，治疗推荐如下（图2）：

**图2:**
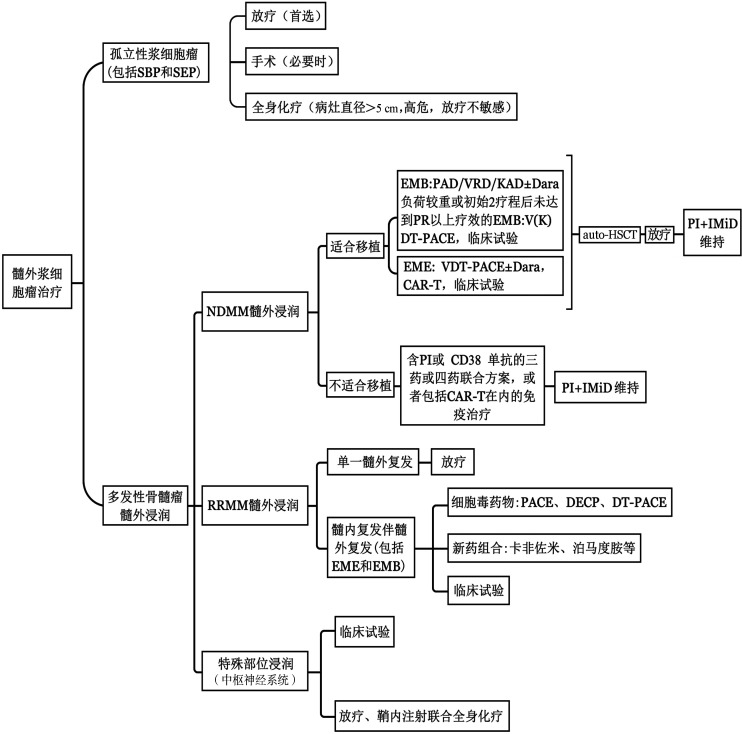
髓外浆细胞瘤的治疗 注 SBP：孤立性骨浆细胞瘤；SEP：孤立性髓外浆细胞瘤；EMB：骨旁浸润；Dara：达雷妥尤单抗；PAD：硼替佐米、阿霉素、地塞米松；VRD：硼替佐米、来那度胺、地塞米松；KAD：卡非佐米、阿霉素、地塞米松；V（K）DT：硼替佐米（卡非佐米）、地塞米松、沙利度胺；PACE：顺铂、阿霉素、环磷酰胺、依托泊苷；EME：非骨旁浸润；PI：蛋白酶体抑制剂；IMiD：免疫调节剂；CAR-T：嵌合抗原受体T细胞；R/R MM：复发/难治多发性骨髓瘤；NDMM：新诊断多发性骨髓瘤；DECP：地塞米松、依托泊苷、环磷酰胺和顺铂

（1）NDMM伴髓外浸润：①适合移植的患者：EMB：推荐含蛋白酶体抑制剂药物的三药或四药联合方案诱导（可联合IMiD）后进行auto-HSCT和维持治疗，并且在移植后联合放疗进一步提高治疗疗效。诱导治疗方案可选择 PAD、VRD、KAD 等，考虑到达雷妥尤单抗可能提高疗效，在诱导治疗方案中可以联合达雷妥尤单抗。或选择含细胞毒药物的诱导方案，如 V（K）DT-PACE等。除此之外，也可考虑临床试验。维持治疗方案推荐使用两药联合方案，如PI+IMiD，维持前是否对髓外病灶行局部放疗目前尚无头对头临床试验。有报道对于治疗后仍有明显肿块或代谢增高的病灶进行放疗可减少复发机会。EME：推荐使用强化的抗骨髓瘤治疗方案［例如达雷妥尤单抗联合VDT-PACE诱导治疗以达到迅速减轻肿瘤负荷的目的，后序贯allo-HSCT和维持治疗。如果有条件，推荐临床试验（如CAR-T）］。②不适合移植的患者：建议EMB或EME患者接受多周期诱导治疗，因不移植患者整体预后较差，诱导方案建议选择含PI和（或）CD38单抗的三药或四药联合方案，或者CAR-T在内的免疫治疗等。若治疗有效，可继续使用有效方案至最大疗效，随后进入维持治疗。

（2）R/R MM 伴髓外浸润：①单一髓外复发：较为罕见，如骨髓明确无受累且病灶单一，可行局部放疗。②髓内伴髓外复发，包括EMB和EME复发，可选择的治疗方案包括：可参考《中国首次复发多发性骨髓瘤诊治指南2022年》[43]相关诊断与治疗原则并同时考虑对髓外病灶有效的治疗方案，如包含卡非佐米或泊马度胺的治疗方案，可选择CAR-T细胞疗效；或细胞毒药物：如PACE（顺铂、阿霉素、环磷酰胺和依托泊苷）、DECP（地塞米松、依托泊苷、环磷酰胺和顺铂）、DT-PACE（地塞米松、沙利度胺、顺铂、阿霉素、环磷酰胺和依托泊苷）；或新型机制药物如塞利尼索等；或进入临床试验。

（3）特殊部位髓外浸润（中枢神经系统浸润）：中枢神经系统浸润MM 患者首先推荐进入临床试验。除此之外，推荐中枢神经系统浸润放疗，鞘内化疗（糖皮质激素、甲氨蝶呤和阿糖胞苷）和基于IMiDs的全身化疗[18]。对于中枢神经系统浸润的患者，全身化疗的困难在于大多数MM化疗药物穿过血脑屏障的渗透性差，但泊马度胺在脑脊液浓度可达39％，可以首选。对于脑实质病变的患者，可进行多学科团队讨论，必要时可考虑手术切除或局部放疗。

五、疗效评估及随访

1. SP疗效评估及随访：尚无针对SP疗效评估的指南，欧洲专家小组提出结合实体瘤（Response Evaluation Criteria in Solid Tumors，RECIST）和IMWG关于MM的疗效标准建立的SP的评估体系，目前并未广泛应用。对于接受放疗的患者，应定期随访并监测实验室及影像学指标；在放疗后的前2年，应每3个月进行1次评估，包括尿液和血清蛋白电泳及免疫固定电泳、血清游离轻链检测、全血细胞计数、血清肌酐和血清钙测定等，2年后若病情稳定可每6个月进行1次。PET-CT（有或无MRI）应在治疗完成后3个月进行，此后每6～12 个月进行1次。必要时行骨髓穿刺术[23]。

2. EMM疗效评估及随访：目前EMM的疗效评估多采用MM和髓外病灶联合评估。MM 疗效评估参照 2016 IMWG 疗效评估标准[44]：包括严格意义的完全缓解、CR、VGPR、PR、微小缓解、疾病稳定、疾病进展、临床复发和CR后复发，并包括微小残留病（minimal residual disease，MRD）评估。

髓外病灶CR的评判要求软组织肿块消失，PR 要求软组织肿块最大垂直径乘积之和缩小>50％[15]。针对MRD阴性，不仅包含了骨髓 MRD 阴性，同时要求原有PET-CT所有高代谢病灶消失。由于EMM患者的血清学反应与髓外疾病的缓解程度之间存在差异，因此必须定期使用影像学方法评估EMM患者的髓外缓解情况。血液学疗效评估应在每个治疗周期开始时进行，而首次通过PET/CT和（或）MRI确定的EMM评估应在启动治疗后 3个月时进行，此后建议1年复检1次。建议基线和随访评估应使用相同的影像学技术。如果怀疑髓外复发或疾病进展，任何时候都可以进行影像学检查。进展的定义为出现新的软组织浆细胞瘤病变或原有1个以上的可测量的软组织病变最大垂直径乘积之和从最低点增加≥50％，或原有的>1 cm的病变长轴增加>50％[15],[44]。
